# Fatty Acids from Pool Lipids as Possible Precursors of the Male Marking Pheromone in Bumblebees

**DOI:** 10.3390/molecules19022330

**Published:** 2014-02-21

**Authors:** Edita Kofroňová, Adam Nekola, Josef Cvačka, Jiří Kindl, Irena Valterová

**Affiliations:** Institute of Organic Chemistry and Biochemistry, Academy of Sciences of the Czech Republic, Flemingovo nám. 2, Prague 166 10, Czech Republic; E-Mails: kofronova@uochb.cas.cz (E.K.); adamnekola@centrum.cz (A.N.); cvacka@uochb.cas.cz (J.C.); kindl@uochb.cas.cz (J.K.)

**Keywords:** *Bombus ruderatus*, *Bombus campestris*, *Bombus bohemicus*, fat body, labial gland secretion, pheromone biosynthesis

## Abstract

Triacylglycerols (TGs) stored in the fat bodies of bumblebee males have a species-specific composition. The striking structural similarities between TG fatty acids (FAs) and components of the male marking pheromone in certain species led to the hypothesis that FAs may serve as precursors in pheromone biosynthesis. Here, we analysed TGs from *B.* *ruderatus*, *B. bohemicus*, and *B. campestris*. Nonadec-9-ene and icos-15-en-1-ol are the main components of *B. ruderatus* labial gland secretion, forming up to 92% of the gland extract. The corresponding icos-11-enic and icos-15-enic acids were found in TGs at levels higher than usual for bumblebee species. We found similar relationships in *B. campestris* and *B. bohemicus*. These results suggest that FAs might be precursors of aliphatic compounds in the male pheromones. Furthermore, we report for the first time the pheromone structure of *B. ruderatus* males.

## 1. Introduction

Most insect species produce pheromones to attract mating partners. However, mating strategies differ widely among insect families. While in Lepidoptera (the best-studied insect family with regard to sex communication) sex pheromones are mostly released by females to attract males for mating, in bumblebees males attract young queens with a pheromone secreted by their labial gland (LG). Males use this pheromone for marking prominent objects on their flight routes (known as patrolling behaviour) [1,2,3]. These marked spots are then attractive for females, *i.e.*, young queens. Components of the labial gland secretion have therefore been called male marking pheromone; however, the term sex pheromone is used by some authors for the same signal [4].

Bioassays performed by Bergman [5] proved that the LG extract of *B. lapidarius* males is behaviourally active and can attract conspecific females. Coppée and co-workers [6] have shown in *B. terrestris* that the attractivity of the LG extract differs with the male age in correlation with the LG secretion quantity and composition [7]. Thorough bioassay showing which components are responsible for the attractiveness of the LG secretion has however not been published. Earlier work showed that the main components of the male LG secretion elicit antennal responses in virgin queens [7] and are thus “hot candidates” for pheromone components. This previous study was performed with two species: *B. terrestris* and *B. lucorum*.

The chemical nature of bumblebee males’ LG secretions has been studied extensively in species occurring in Europe [4,8,9,10,11,12,13,14,15,16]. Each species produces a specific blend of compounds [17,18]. The gland secretion primarily contains two types of compounds: straight-chain aliphatic alcohols, aldehydes, and esters as one type and isoprenoid compounds as the second type.

Little is known about pheromone biosynthesis in bumblebees as opposed to moths, where biosynthetic pathways are well-described and understood [19,20,21]. There are two potential metabolic pathways leading to aliphatic compounds: biosynthesis from common lipids in the body or *de novo* synthesis from acetate units [22]. The first paper discussing the biosynthesis of marking pheromone was based on analysis of compounds isolated from 22 bumblebee species [23]. The authors suggested that these compounds are produced from saturated FAs by the action of specific glandular desaturases. Considering the recent literature and our own experiments, it seems that both pathways are possible. Most experiments have been performed with three model species: *B. terrestris*, *B. lucorum*, and *B. lapidarius*. While *B. lucorum* and *B. lapidarius* produce only aliphatic compounds in their pheromone glands [1,8,13], *B. terrestris* produces aliphatic and terpenic alcohols and esters [7,8]. *De novo* biosynthesis of pheromone components was demonstrated by *in vitro* experiments in two species, *B. terrestris* and *B. lucorum* [24]. Incubations of labial glands with ^14^C-labelled acetate led to formation of FAs, esters (both species), and terpenoids (*B. terrestris*). On the other hand, a large series of experiments with deuterium-labelled FAs of different chain lengths applied *in vivo* or administered with food gave rise to aliphatic alcohols (*B. lapidarius*) and fatty acid ethyl esters (*B. lucorum*) in the LGs, as well as labelled triacylglycerols (TGs) in the fat bodies (FBs) [25,26]. These results support the hypothesis that lipidic precursors are involved in pheromone biosynthesis.

TGs stored in the FBs of bumblebee males have species-specific compositions [27]. Both the amount and composition of TG undergo changes with male age and these changes correlate with the dynamics of the marking pheromone production (study in *B. terrestris* and *B. lucorum*) [28]. In some cases, striking structural similarity between the TG FAs and components of the male marking pheromone supports the hypothesis that FAs may serve as precursors in pheromone biosynthesis. Such similarities can be seen in *B. pratorum* [8,29], *B. lapidarius* [1,30], and *B. confusus* [11,30]. In *B. pratorum*, this similarity is particularly conspicuous; two major aliphatic pheromone components, octadec-11-en-1-ol (15% in the LG) and pentacosa-7,17-diene (7% in the LG), may be formed in the LG from octadec-11-enoic acid (15% in the FB) and hexacosa-9,19-dienoic acid (5% in the FB), respectively, by a simple structural modification [29]. The aim of this study is to search for other bumblebee species in which structural similarity between FAs and pheromone components supports the lipid precursor hypothesis for the biosynthesis of male marking pheromone. Specifically, we studied three species in detail: *Bombus (Megabombus) ruderatus*, *B. (Metapsithyrus) campestris*, and *B. (Ashtonipsithyrus) bohemicus.* Furthermore, chemical analysis of the LG secretion of *B. ruderatus* males and detailed analyses of TGs and FAs in the FBs of all three species are reported here for the first time.

## 2. Results and Discussion

**Figure 1:**
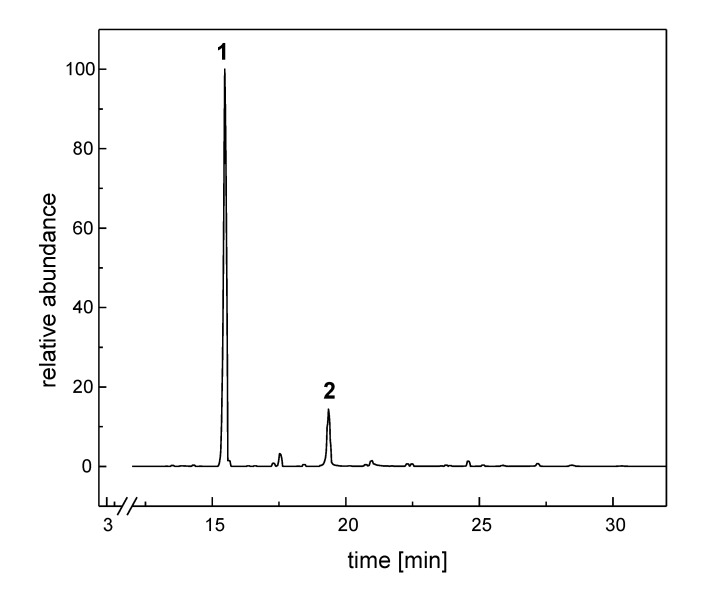
Gas chromatogram of the labial gland extract of *B. ruderatus* males; **1**—nonadec-9-ene; **2**—icos-15-en-1-ol.

In the LG extract of *B. ruderatus* males (14 specimens), we identified 36 compounds. The secretion consisted of two major components and a number of medium and minor ones (Figure 1). Components present in amounts exceeding 0.1% (17 compounds) are shown in Table 1. Nonadec-9-ene dominated in the mixture (77%), followed by icos-15-en-1-ol (15%). Except for farnesol and its two esters, the secretion consisted of aliphatic compounds, mainly hydrocarbons.

Saturated and unsaturated straight-chain hydrocarbons are always present in the LG extracts of bumblebee males. Their content usually ranges between 3%–10% with oxygenated compounds being the main secretion components. In *B. ruderatus*, however, nonadec-9-ene is the main secretion component (77%), and it most probably plays a role in the chemical communication as has been shown earlier in other species for main LG components [7]. The presence of nonadec-9-ene as a component of male LG secretion seems to be typical for the whole subgenus *Megabombus*. While no species from the many other subgenera studied to date produce hydrocarbons as a main LG component, all *Megabombus* species have nonadec-9-ene as a major or medium component: *B. hortorum*, 20% [8,31]; *B. gerstackeri*, 26% [32]; *B. consobrinus*, 42% [32]; *B. portschinski*, 46% [33]; and *B. argilaceus*, 84% [33]. The occurrence of nonadec-9-ene seems to be a common trait in *Megabombus*, indicating that these species share a common biosynthetic pathway for pheromone formation.

**Table 1:** Composition (relative percent) of the labial gland secretion of *B. ruderatus* males (N=14). Components < 0.1% are not listed.

Compound	Mean (%)	Median (%)	Standard deviation (%)
(*E*,*E*)-Farnesol	0.7	0.02	1.4
Octadec-9-ene	0.2	0.1	0.2
Nonadec-9-ene	76.7	78.0	8.4
Nonadecane	0.9	0.6	0.5
Henicos-9-ene	0.4	0.2	0.4
Henicosane	1.2	0.8	0.8
Icos-15-en-1-ol	15.0	16.4	8.3
Tricosane	3.6	3.1	2.4
Pentacos-9-ene	0.1	0.07	0.2
Pentacosane	0.4	0.2	0.3
Docosen-1-ol ^a^	0.1	0.0	0.1
Heptacos-9-ene	0.2	0.1	0.4
Heptacosane	0.1	0.1	0.1
Farnesyl tetradecenoate ^b^	0.4	0.07	0.7
Hentriacont-9-ene	0.1	0.1	0.1
Farnesyl octadecadienoate	0.2	0.1	0.3
Icosenyl tetradecenoate ^c^	0.2	0.05	0.5

^a^ Unseparable mixture of docos-15-en-1-ol and docos-17-en-1-ol; ^b^ Unseparable mixture of 2 isomers; ^c^ Double bond positions not determined (DMDS adducts not found).

**Figure 2:**
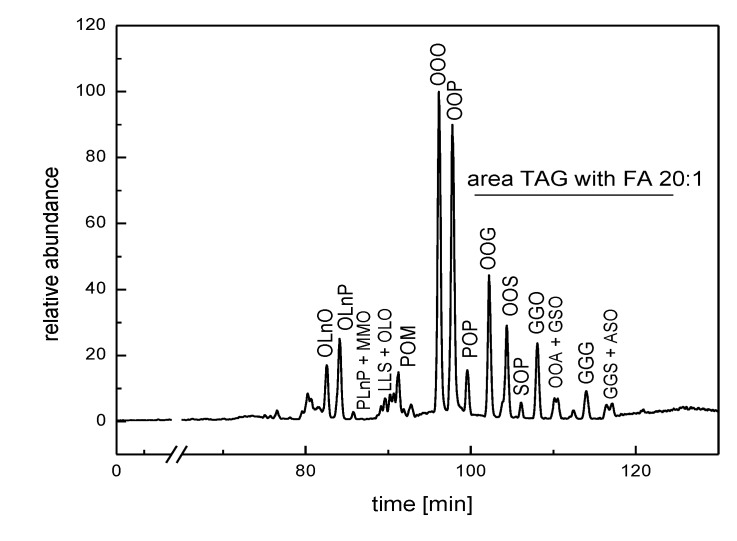
HPLC chromatogram of TG fraction of the fat body extract of *B. ruderatus* males. FA residues in TGs are abbreviated as follows (in alphabetic order): arachidic (A), gondoic (G), linoleic (L), α-linolenic (Ln), myristic (M), oleic (O), palmitic (P), and stearic (S).

More than 60 different TGs have been identified in the TG fraction of FB lipid extract from *B. ruderatus* males (Table 2 and [27]). Similar to TGs from other bumblebee species, *B. ruderatus* TGs have 18:1 FA in highest abundance (TG 16:0_18:1_18:1 and TG 18:1_18:1_18:1). The retention order of TGs usually follows the equivalent carbon number, ECN (ECN=CN−2DB, where CN is the number of carbon atoms and DB is the number of double bonds in a molecule). The ECN values for *B. ruderatus* TGs ranged between 40 and 60 (Table 2). In TGs with ECN>50, 20:1 FA was highly abundant (11% of TGs contained the 20:1 moiety; Table 2, Figure 2).

**Table 2:** Composition of triacylglycerols in the fat body of *B. ruderatus* males.

ECN	CN:DB	Triacylglycerol (TG) ^a^	Peak area relative % (mean ± SD; N = 4)
40	48:4	18:3_18:1_12:0; 18:3_14:0_16:1	0.1 ± 0.1
	46:3	16:0_18:3_12:0; 14:0_14:0_18:3	0.1 ± 0.1
42	54:6	18:1_18:3_18:2; 18:2_18:2_18:2	0.2 ± 0.1
	52:5	18:3_18:1_16:1; 18:2_18:2_16:1; 18:2_16:0_18:3	0.8 ± 0.2
	50:4	18:3_18:1_14:0; 18:3_16:0_16:1	0.8 ± 0.2
	48:3	16:0_18:3_14:0; 18:2_18:1_12:0; 18:2_14:0_16:1	0.4 ± 0.4
	46:2	16:1_18:1_12:0; 16:1_16:1_14:0	0.1 ± 0.1
	44:1	14:0_18:1_12:0; 14:0_16:1_14:0	0.1 ± 0.1
44	54:5	18:1_18:3_18:1	3.3 ± 1.8
	52:4	18:1_18:3_16:0	5.1 ± 2.1
	50:3	16:0_18:3_16:0; 18:2_16:1_16:0	1.6 ± 0.2
	48:2	18:1_18:1_12:0; 18:1_16:1_14:0; 16:1_16:1_16:0	1.3 ± 0.5
	46:1	16:0_18:1_12:0; 14:0_14:0_18:1	0.7 ± 0.4
46	56.5	18:1_18:1_20:3	0.7 ± 0.3
	54:4	18:2_18:2_18:0; 18:1_18:1_18:2	2.2 ± 0.2
	52:3	18:2_18:1_16:0; 18:2_18:0_16:1; 18:1_18:1_16:1	7.1 ± 1.6
	50:2	18:1_18:1_14:0; 18:1_16:1_16:0	6.6 ± 3.2
	48:1	16:0_18:1_14:0	2.4 ± 1.4
48	58:5	18:2_18:2_22:1	0.6 ± 0.1
	56.4	18:1_18:1_20:2	0.5 ± 0.2
	54:3	18:1_18:1_18:1	19.4 ± 2.5
	52:2	18:1_18:1_16:0	22.8 ± 4.7
	50:1	16:0_18:1_16:0	4.0 ± 1.6
50	56:3	18:1_18:1_20:1	4.9 ± 2.5
	54:2	18:1_18:1_18:0	5.0 ± 0.6
	52:1	18:0_18:1_16:0	1.4 ± 0.5
52	58:3	20:1_20:1_18:1; 18:1_22:1_18:1	2.5 ± 1.7
	56:2	20:1_18:0_18:1; 18:1_18:1_20:0	1.1 ± 0.7
	54:1	20:0_16:0_18:1; 18:0_18:0_18:1	0.5 ± 0.1
54	60:3	20:1_20:1_20:1; 20:1_22:1_18:1; 18:1_18:1_24:1	0.9 ± 0.8
	58:2	20:1_20:1_18:0; 18:1_18:1_22:0	0.9 ± 0.7
	56:1	20:0_18:1_18:0	0.2 ± 0.1
56	62:3	18:1_18:1_26:1	0.5 ± 0.5
	60:2	18:1_18:1_24:0	0.4 ± 0.3
	58:1	22:0_18:1_18:0; 18:0_26:1_14:0; 18:0_24:0_16:1	0.1 ± 0.1
58	64:3	18:1_18:1_28:1	0.2 ± 0.2
	62:2	22:1_20:0_20:1; 18:1_18:1_26:0	0.2 ± 0.2
60	66:3	18:1_18:1_30:1	0.2 ± 0.2
	64:2	18:1_18:1_28:0	0.2 ± 0.1

^a^ Shorthand notation of TGs according to Liebisch and co-workers [34]. The most likely FA in the *sn*-2 position of the glycerol is presented as second acid in the notation [28].

The highest content of 20:1 FA was in TGs bearing 18:1 or 18:0 acids, such as TG 18:1_18:1_20:1, TG 18:1_20:1_20:1, and TG 18:1_18:0_20:1. A TG with three 20:1 moieties, *i.e.*, TG 20:1_20:1_20:1, was also detected. The relative amount of 20:1 acid methyl ester in the transesterified sample was 3.3% (Table 3). This amount is relatively high compared to those found in other, previously studied bumblebee species (usually below 1% if present at all) [30].

**Table 3:** Relative proportions (in %) of fatty acids in TG identified after a transesterification of TG fraction from *B. ruderatus*, *B. campestris*, and *B. bohemicus* fat body extracts.

Fatty acid (Trivial name)	Code	*Bombus* species		
		*ruderatus*	*campestris*	*bohemicus*
Decanoic (Capric)	10:0	0.3	trace	0.1
Dodecanoic (Lauric)	12:0	-	0.4	10.1
Tetradec-9-enoic (Myristoleic)	9-14:1	0.2	0.1 ^a^	6.0
Tetradecanoic (Myristic)	14:0	1.3	1.6	15.6
Hexadec-9-enoic (Palmitoleic)	9-16:1	5.1	1.2	14.3
Hexadec-11-enoic (Palmitvaccenic)	11-16:1	1.1	-	2.3
Hexadecanoic (Palmitic)	16:0	14.9	15.3	22.3
Octadecadienoic ^a^	18:2	0.1	2.3	2.5
Octadec-9-enoic (Oleic)	9-18:1	68.5	56.1	23.5
Octadec-11-enoic (*cis*-Vaccenic)	11-18:1	-	4.3	1.2
Octadec-13-enoic (No trivial name)	13-18:1	trace	-	0.1 ^a^
Octadecanoic (Stearic)	18:0	4.1	12.6	1.6
Icosadienoic ^a^	20:2	-	-	trace
Icos-11-enoic (Gondoic)	11-20:1	3.3	1.7	0.1 ^a^
Icos-15-enoic (No trivial name)	15-20:1	trace	-	-
Icosanoic (Arachidic)	20:0	0.5	1.4	0.1
Docos-13-enoic (Erucic)	13-22:1	0.5	-	-
Docosanoic (Behenic)	22:0	0.1	0.4	-
Tetracosenoic ^a^	24:1	0.1	-	-
Tetracosanoic (Lignoceric)	24:0	trace	0.2	-

^a^ Double bond position not determined (the diagnostic CI-MS fragments not found).

To investigate a possible connection with pheromone biosynthesis, we determined the double bond position in 20:1 methyl ester using chemical ionisation mass spectrometry with acetonitrile as reaction gas [35]. The presence of diagnostic fragments *m/z* 208 and *m/z* 280 indicated the presence of a double bond in position 11 (gondoic acid methyl ester). Formation of nonadec-9-ene from gondoic acid via decarboxylation is a potential pathway to pheromone biosynthesis (Scheme S1) [19].

Another isomer of 20:1 methyl ester was present in the transesterified sample in a much smaller proportion. The CI spectrum showed fragments *m/z* 152 and *m/z* 336, corresponding to a double bond at position 15 in the 20:1 methyl ester. The chromatographic separation of the Δ11 and Δ15 isomers was, however, poor. The small peak corresponding to the Δ15 isomer was nearly hidden under the peak of the prevailing Δ11 isomer. Thus, the diagnostic fragments in CI-MS of the Δ15 isomer were not as pronounced as those in the spectrum of the Δ11 isomer. Formation of alcohols from fatty acids by the action of specific glandular fatty-acyl reductases has been proposed [36]. Icos-15-en-1-ol is present in the LG secretion in a lesser amount than nonadec-9-ene (1:5 ratio), which correlates with the excess of the Δ11 isomer of 20:1 acid that we observed in TGs.

Since nonadec-9-ene occurs in all *Megabombus* species studied so far, it would be interesting to investigate the lipid composition in more species of this subgenus to see whether the similarity between lipids and pheromone components found in *B. ruderatus* is a general pattern in *Megabombus*. To date, *B. hortorum* is the only other *Megabombus* species for which an analysis of TGs has been reported. The proportion of gondoic acid in the TG fraction of *B. hortorum* was rather low (0.7%). *B. hortorum*, however, produces a rather complex mixture in the LG secretion [8], in which both aliphatic and terpenic compounds are present. Thus, the enzymatic biosynthetic system in *B. hortorum* is also likely rather complex.

Encouraged by the results with *B. ruderatus*, we focused on other bumblebee and cuckoo-bumblebee species that produce aliphatic compounds as their major LG secretion components. Of the species from which we had previously analysed LG secretions, we selected two as candidates for a detailed study of TG composition and FA structure: *B. campestris* and *B. bohemicus.* The LG secretion of *B. campestris* males contains icos-11-en-1-ol (21%) and its corresponding acetate (1.3%), icos-11-enal (9%), octadec-11-enal (25%), and octadec-11-en-1-ol (7%) and its acetate (1%) [14]. If the lipid precursor hypothesis holds true (Scheme S1), we would expect to see high levels of icos-11-enoic (gondoic) acid and octadec-11-enoic (*cis*-vaccenic) acid in TGs (Table 4). In fact, the relative amounts were 4.3% *cis*-vaccenic acid and 1.7% gondoic acid (Table 3). Considering that the main role of TGs in the FBs is to provide a pool of energy mostly for the flight muscles, the amount of specific FAs available for the production of secondary metabolites such as pheromones is quite substantial. Furthermore, Tomčala and co-workers [37] showed in *Locusta migratoria* that FB FAs with chains longer than 18 carbons are not mobilised in response to adipokinetic hormone application. Thus, longer chain FAs might play roles other than participation in primary metabolism and energy supply in bumblebees as well.

The LG secretion of *B. bohemicus* males is dominated by hexadec-11-en-1-ol (65%) and the corresponding hexadec-11-enal (5%) [14,38]. Octadec-11-en-1-ol and octadec-13-en-1-ol, together with the corresponding aldehydes, form minor secretion components (altogether 3%). The C_20_ chain is of medium abundance and is mostly represented by icosa-11,15-dienal (14%) with minor amounts of monounsaturated C_20_ derivatives such as icosa-11-en-1-ol, icosa-15-en-1-ol, and the corresponding icosenals, forming altogether 2% of the secretion mixture. Most of the expected FAs were found in FB TGs (Table 3 and Table 5) in various quantities. Palmitvaccenic (11-16:1) and *cis*-vaccenic (11-18:1) acids together formed 4% of the FA mixture. Almost 23% of TGs bore 20:1 and/or 20:2 FAs (Table 5).

**Table 4:** Composition of triacylglycerols in the fat body of *B. campestris* males.

ECN	CN:DB	Triacylglycerol (TG) ^a^	Peak area relative % (mean ± SD; N = 5)
40	54:7	18:2_18:3_18:2; 18:3_18:3_18:1	0.1 ± 0.1
	52:6	18:2_18:3_16:1; 18:3_18:3_16:0	0.1 ± 0.1
	48:4	12:0_18:2_18:2	0.1 ± 0.0
42	54:6	18:2_18:2_18:2; 18:3_18:1_18:2	0.2 ± 0.2
	52:5	18:2_18:2_16:1; 16:1_18:3_18:1	0.3 ± 0.2
	50:4	14:0_18:2_18:2; 18:1_14:0_18:3	0.3 ± 0.1
	48:3	16:1_16:1_16:1; 16:0_18:3_14:0; 18:2_14:0_16:1	0.1 ± 0.0
44	54:5	18:1_18:3_18:1	7.4 ± 4.0
	52:4	18:1_18:3_16:0	6.1 ± 2.4
	50:3	16:1_14:0_20:2; 16:1_16:1_18:1; 16:0_16:1_18:2	0.2 ± 0.1
	48:2	18:1_18:1_12:0	0.4 ± 0.1
	46:1	12:0_18:1_16:0; 14:0_14:0_18:1; 16:0_16:1_14:0	0.3 ± 0.1
46	56:5	18:2_18:2_20:1; 18:1_18:1_20:3; 18:1_18:2_20:2	0.7 ± 0.2
	54:4	18:1_18:1_18:2; 18:3_18:1_18:0	3.8 ± 0.7
	52:3	16:1_18:1_18:1; 16:0_18:1_18:2	2.7 ± 0.2
	50:2	18:1_14:0_18:1; 16:0_18:1_16:1	3.4 ± 0.4
	48:1	16:0_16:1_16:0; 14:0_18:1_16:0	0.6 ± 0.2
48	54:3	18:1_18:1_18:1	24.5 ± 2.3
	52:2	16:0_18:1_18:1	27.4 ± 2.3
	50:1	16:0_18:1_16:0; 14:0_18:0_18:1	1.3 ± 0.5
50	56:3	18:1_20:1_18:1	2.1 ± 1.0
	54:2	18:1_18:0_18:1; 18:0_18:2_18:0	11.6 ± 2.3
	52:1	16:0_18:1_18:0; 18:0_16:1_18:0	2.7 ± 1.2
52	58:3	18:1_20:1_20:1	0.1 ± 0.1
	56:2	18:1_20:0_18:1	1.6 ± 0.7
	54:1	18:0_18:0_18:1	1.1 ± 0.5

^a^ Shorthand notation of TGs according to Liebisch and co-workers [34]. The most likely FA in the *sn*-2 position of the glycerol is presented as second acid in the notation [28].

Enzymes catalysing reduction of fatty acids (FARs) in bumblebees would certainly deserve investigation in the future. So far, not many studies are devoted to insect reductases [36]. Most of known FARs catalyse FAs reduction to alcohols, aldehydes being intermediates of this reaction. In bumblebee labial glands however, aldehydes occur in high amounts in some species. Thus, some of aldehyde-generating FARs can be expected to operate in the male labial glands. Such enzymes were so far isolated only from plants or algae [39].

Earlier studies of TGs in lipids of male bumblebees were focused on the distribution of different TGs with regard to species-specificity. Intact TGs have been analysed and reported [27] without transesterification and determination of double bond positions. Here, we report these details for three species (one bumblebee and two cuckoo-bumblebee species), showing that species-specific differences also occur on the level of FA structures.

**Table 5:** Composition of triacylglycerols in the fat body of *B. bohemicus* males.

ECN	CN:DB	Triacylglycerol (TG) ^a^	Peak area relative % (mean ± SD; N = 6)
38	54:8	18:3_18:3_18:2	0.1 ± 0.0
	52:7	16:1_18:3_18:3	0.2 ± 0.0
	50:6	14:0_18:3_18:3	0.2 ± 0.0
	48:5	14:1_16.1_18:3	0.1 ± 0.0
	46:4	12:0_16:1_18:3	0.2 ± 0.1
	44:3	14:0_12:0_18:3	0.2 ± 0.1
40	54:7	18.3_18:1_18:3; 18:2_18:3_18:2	1.0 ± 0.3
	52:6	18:3_18:3_16:0	1.0 ± 0.3
	50:5	16:1_16:1_18:3	0.8 ± 0.2
	48:4	12:0_18:3_18:1;	1.9 ± 0.7
	46:3	16:1_16:1_14:1; 16:0_18:3_12:0	0.9 ± 0.3
	44:2	16:1_16:1_12:0	0.2 ± 0.1
42	54:6	18:2_18:2_18:2; 18:3_18:1_18:2	1.3 ± 0.3
	52:5	18:2_18:2_16:1; 16:1_18:3_18:1	4.5 ± 0.5
	50:4	14:0_18:2_18:2; 16:1_16:1_18:2; 18:3_16:0_16:1	5.6 ± 1.0
	48:3	16:1_16:1_16:1; 14:0_14:1_20:2; 14:0_16:0_18:3	2.5 ± 0.9
	46:2	16:1_18:1_12:0; 16:1_16:1_14:0	1.1 ± 0.6
	44:1	12:0_18:1_14:0; 14:0_16:1_14:0; 12:0_16:1_16:0	0.9 ± 0.4
44	54:5	18:2_18:1_18:2; 18:1_18:3_18:1	9.4 ± 4.2
	52:4	18:3_18:1_16:0; 18:2_18:2_16:0; 16:1_16:1_20:2	12.0 ± 3.2
	50:3	16:1_16:1_18:1; 14:0_16:0_20:2; 16:0_16:0_18:3	4.6 ± 1.4
	48:2	18:1_18:1_12:0; 18:1_16:1_14:0; 16:1_16:1_16:0	4.1 ± 1.4
	46:1	12:0_18:1_16:0; 14:0_14:0_18:1; 16:0_16:1_14:0	2.8 ± 0.9
46	56:5	20:2_16:1_20:2; 18:1_18:1_20:3	0.3 ± 0.3
	54:4	18:1_18:1_18:2; 18:2_16:0_20:2	3.6 ± 1.1
	52:3	16:1_18:1_18:1; 16:0_18:1_18:2	6.5 ± 0.5
	50:2	18:1_14:0_18:1; 16:0_18:1_16:1	8.1 ± 2.0
	48:1	16:0_16:1_16:0; 14:0_18:1_16:0	3.5 ± 1.6
48	54:3	18:1_18:1_18:1	7.1 ± 2.9
	52:2	16:0_18:1_18:1	9.7 ± 0.9
	50:1	16:0_18:1_16:0	2.6 ± 1.2
50	56:3	18:1_18:1_20:1; 18.2_20:0_18:1	0.2 ± 0.2
	54:2	18:1_18:0_18:1; 18:0_18:2_18:0	1.3 ± 0.3
	52:1	16:0_18:1_18:0; 18:0_16:1_18:0	0.6 ± 0.3
52	58:3	18:1_18:1_22:1	0.1 ± 0.1
	56:2	18:1_18:1_20:0	0.2 ± 0.1
	54:1	18:0_18:0_18:1; 20:0_16:0_18:1	0.2 ± 0.1

^a^ Shorthand notation of TGs according to Liebisch and co-workers [34]. The most likely FA in the *sn*-2 position of the glycerol is presented as second acid in the notation [28].

## 3. Experimental

### 3.1. Biological Materials

*Bombus (Megabombus) ruderatus* (Fabricius, 1775) males were obtained from laboratory rearing. Colonies were started with females collected in the region of Eastern Prague and settled in the laboratory. When colonies turned to male production, the male imagoes were collected immediately after emergence to avoid possible inbreeding. They were placed in a plastic box together with several workers and supplied with high-quality food. The temperature was kept between 28.5–29.5 °C. For chemical analyses, males were killed by freezing at −18 °C during the photophase prior to dissection of the tissues.

The males of *Bombus (Ashtonipsithyrus) bohemicus* originated from an invaded colony of *Bombus* *lucorum*, where the workers were allowed to collect food from nature. *Bombus (Metapsithyrus)* *campestris* (Panzer, 1801) males were collected in the Krkonoše Mountains (northeastern Czech Republic, elevation 750–800 m). For chemical analyses, the collected living insects were transported to the laboratory and then stored in a freezer until the fat bodies were dissected. The insect material is deposited in the collection of one of the authors (J.K.).

### 3.2. Sample Preparation

Males of productive age (5–20 days old) were taken from the rearing (*B. ruderatus*, 14 specimens) or collected in the open air (*B. campestris*, nine specimens). The labial glands were dissected and extracted with hexane (200 µL per gland). Dissected fat bodies were extracted with chloroform/methanol as previously described [30]. After pre-separation of the lipid extracts using TLC (mobile phase—hexane:diethyl ether:formic acid, 80:20:1), the TG fraction was stored in chloroform solution (0.1 mg/10 µL) [30] at −18 °C prior to analysis. Both labial glands and fat bodies of *B. ruderatus* were dissected, while only the fat bodies of *B. campestris* and *B. bohemicus* were dissected and analysed in this work. The data obtained from lipid analyses were compared with previously published analytical data for LG secretions from *B. campestris* and *B. bohemicus* males [14].

### 3.3. GC/MS Analysis of the Labial Gland Extracts

The extracts were analysed using a gas chromatograph with a splitless injector (220 °C) and mass detector (200 °C, Fisons MD 800 coupled to a Focus GC, Thermo Finnigan S.p.A., Milan, Italy). A DB-5ms column (30 m × 0.25 mm, film thickness 0.25 µm, Agilent Technologies) and helium gas (constant flow of 1 mL/min) were used for separations. The temperature program started at 70 °C, after 2 min delay the temperature of the oven was increased to 140 °C at a rate of 40 °C/min, then to 240 °C at a rate of 2 °C/min, and finally to 320 °C (15 min delay) at a rate of 4 °C/min. Compounds were identified based on their mass spectra as compared to spectra in the National Institute of Standards and Technology Library (NIST, city, state abbrev, USA). Positions of double bonds in unsaturated LG extract components were determined based on preparation of dimethyl disulfide adducts followed by GC/MS analysis [40]. The products were analysed by GC/MS using the same temperature program as for the original extracts.

### 3.4. HPLC/APCI-MS Analysis of TGs

The HPLC/MS analyses were performed on a system comprising an SMC 1000 vacuum membrane degasser, P 4000 gradient pump, SN 4000 control unit (Thermo Separation Products), and LCQ classic ion-trap mass spectrometer (Thermo Finnigan). Manual injection was accomplished with a Rheodyne type model D injection valve (Ecom, Prague, Czech Republic) equipped with a 5-μL internal injection loop. Two stainless steel Nova-Pak C18 columns (300 × 3.9 mm, 150 × 3.9 mm, particle size 4 μm; Waters) were connected in series and kept at 30 °C. The chromatographic conditions used were developed previously [28] and optimised for bumblebee TG separations [41]. Briefly, non-aqueous reversed phase HPLC gradient separations were performed using acetonitrile (A) and 2-propanol (B) in the mobile phase (gradient: 0 min—100% A, flow rate of 1 mL/min; 108 min—30% A, 70% B, 1 mL/min; 150 min—5% A, 95% B, 0.5 mL/min; 165 min—5% A, 95% B, 0.5 mL/min; 177 min—100% A, 0.5 mL/min; 180 min—100% A, 1 mL/min). Ammonium acetate dissolved in 2-propanol/water (1:1) to a final concentration of 100 mM was added post-column at a flow rate of 10 μL/min. The APCI source was operated at 400 °C, the heated capillary temperature was 200 °C, and the corona discharge current was set to 4.5 μA. The full scan mass spectra were recorded in the *m/z* range of 75–1,300. Mass spectra were interpreted with the aid of *TriglyAPCI* software [42]. The peak areas were calculated from reconstructed chromatograms for the [M+H]^+^ and [M+NH_4_]^+^ ions (both summed together).

### 3.5. Transesterification of TG and GC/MS Analysis of FAMEs

The TG fraction from TLC separation was transesterified using acetylchloride in chloroform/methanol as previously described [43]. The reaction mixture was neutralized with silver carbonate. After a brief centrifugation, the supernatant was directly injected into GC/MS, and the resulting FAMEs were identified. Positions of double bonds in unsaturated FAMEs were determined from chemical ionisation mass spectra (reaction gas: acetonitrile) recorded on an ion trap mass analyser (Varian Saturn 2000R, Varian, Walnut Creek, CA, USA) as described in the literature [35].

## 4. Conclusions

Our results show that specific FAs bound in TGs of the male fat body are structurally similar to the major aliphatic components of the male marking pheromone in one bumblebee and two cuckoo-bumblebee species. Thus, our results support the hypothesis that the pheromonal components may be formed from lipidic precursors after their transport to and structural modification in the labial gland. Putting together all results obtained to date, it is likely that both pathways (*de novo* and transport pathways) are active in the organism, and the dominant one may depend on the physiological state of the animal or the availability of particular precursors.

## Abbreviations

APCI-MS: atmospheric pressure chemical ionisation mass spectrometry
CI: chemical ionisation
CN: number of carbon atoms
DB: number of double bonds
ECN: equivalent carbon number
FA: fatty acid
FAME: fatty acid methyl ester
FAR: fatty acyl-CoA reductase
FB: fat body
GC/MS: gas chromatography and mass spectrometry
HPLC: high performance liquid chromatography
LG: labial gland
SD: standard deviation
TG: triacylglycerol

## Supplementary Materials

Supplementary materials can be accessed at: http://www.mdpi.com/1420-3049/19/2/2330/s1.
