# Supplementary material for: Fatty Acids from Pool Lipids as Possible Precursors of the Male Marking Pheromone in Bumblebees
Source: Molecules. 2014 Feb 21;19(2):2330–43. doi: 10.3390/molecules19022330 (PMC6271375; doi:10.3390/molecules19022330)

Supplementary Materials

Scheme S1. Putative biosynthetic pathways of main components of the labial gland secretion of *Bombus ruderatus*, *B. campestris*, and *B. bohemicus* males.

Bombus ruderatus

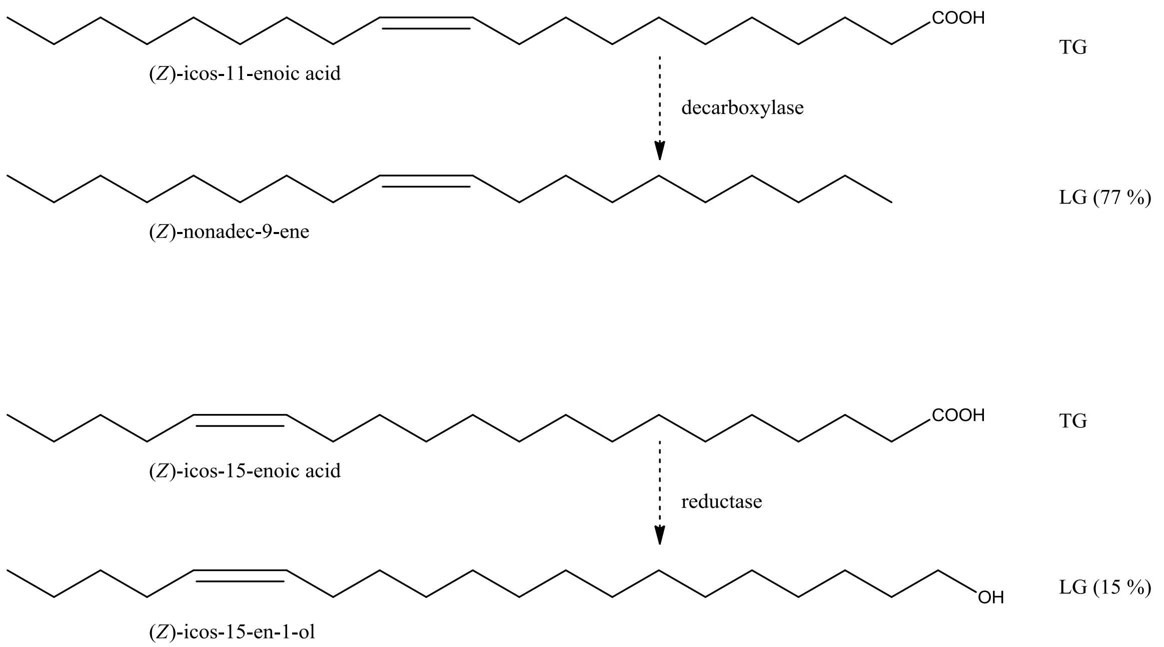

Bombus campestris

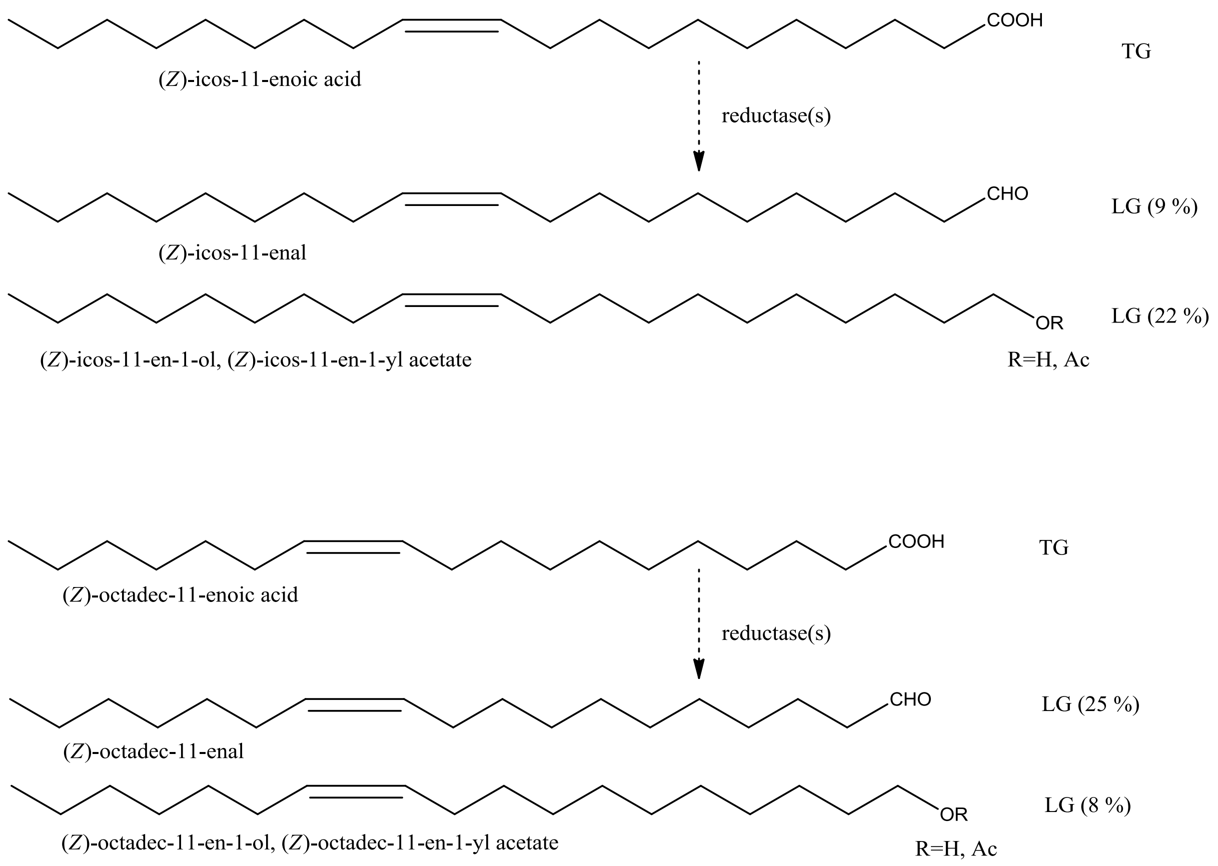

Scheme S1. *Cont.*

Bombus bohemicus

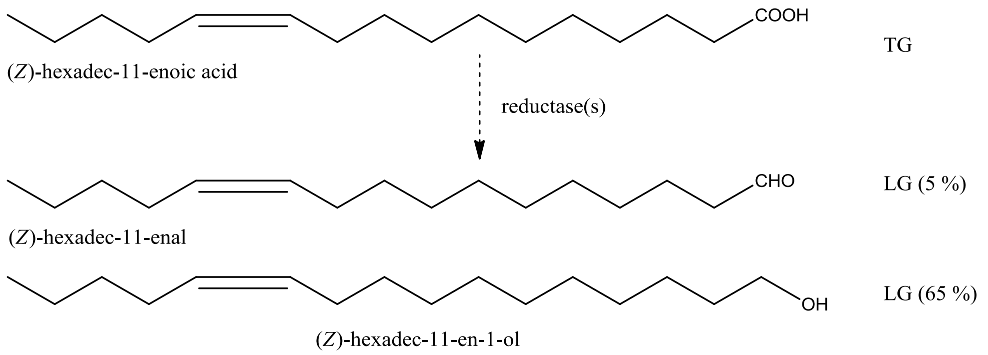

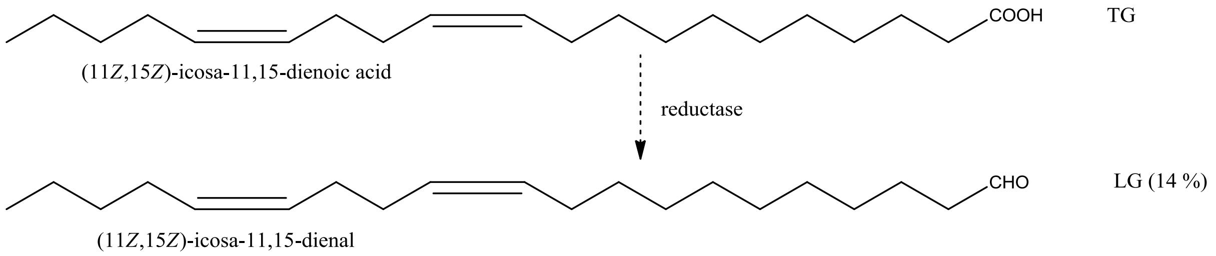

Supplement: Supplementary file 1 [file molecules-19-02330-s001.pdf]
